# A secondary role for hypoxia and HIF1 in the regulation of (IFNγ-induced) PD-L1 expression in melanoma

**DOI:** 10.1007/s00262-021-03007-1

**Published:** 2021-07-15

**Authors:** Anneloes van Duijn, Karin J. Willemsen, Nathalie O. P. van Uden, Lieke Hoyng, Sterre Erades, Jan Koster, Rosalie M. Luiten, Walbert J. Bakker

**Affiliations:** 1 Laboratory of Experimental Dermatology, Department of Dermatology and Netherlands Institute for Pigment Disorders, Amsterdam Infection & Immunity Institute, Amsterdam University Medical Centers, University of Amsterdam, Cancer Center Amsterdam, Meibergdreef 9, 1105 AZ Amsterdam, The Netherlands; 2 Department of Oncogenomics, Amsterdam University Medical Centers, University of Amsterdam, Meibergdreef 9, 1105 AZ Amsterdam, The Netherlands

**Keywords:** PD-L1, Hypoxia, Melanoma, HIF1, IFNγ, Immunotherapy

## Abstract

Cancer cells are able to escape immune surveillance by upregulating programmed death ligand 1 (PD-L1). A key regulator of PD-L1 expression is transcriptional stimulation by the IFNγ/JAK/STAT pathway. Recent studies suggest that hypoxia can induce PD-L1 expression. As hypoxia presents a hallmark of solid tumor development, hypoxic control of PD-L1 expression may affect the efficacy of cancer immunotherapy. This study aims to explore the hypoxic regulation of PD-L1 expression in human melanoma, and its interaction with IFNγ-induced PD-L1 expression. Analysis of the cutaneous melanoma dataset from the cancer genome atlas revealed a significant correlation of the HIF1-signaling geneset signature with *PD-L1* mRNA expression. However, this correlation is less pronounced than other key pathways known to control PD-L1 expression, including the IFNγ/JAK/STAT pathway. This secondary role of HIF1 in PD-L1 regulation was confirmed by analyzing single-cell RNA-sequencing data of 33 human melanoma tissues. Interestingly, PD-L1 expression in these melanoma tissues was primarily found in macrophages. However, also in these cells STAT1, and not HIF1, displayed the most pronounced correlation with *PD-L1* expression. Moreover, we observed that hypoxia differentially affects PD-L1 expression in human melanoma cell lines. Knockdown of HIF1 expression indicated a minor role for HIF1 in regulating PD-L1 expression. A more pronounced influence of hypoxia was found on IFNγ-induced *PD-L1* mRNA expression, which is controlled at a 952 bp *PD-L1* promoter fragment. These findings, showing the influence of hypoxia on IFNγ-induced PD-L1 expression, are relevant for immunotherapy, as both IFNγ and hypoxia are frequently present in the tumor microenvironment.

**Supplementary Information:**

The online version contains supplementary material available at 10.1007/s00262-021-03007-1.

## Introduction

Immune checkpoint inhibitors (ICI) targeting programmed cell death 1 (PD-1) have significantly improved the survival outcome of melanoma [1]. PD-1 is expressed by various immune cells, including tumor-infiltrating lymphocytes, and binds to programmed cell death ligand 1 (PD-L1) [1, 2]. PD-L1 is constitutively expressed by T and B lymphocytes, whereas its expression can be induced in non-immune cells, including cancer cells [3]. PD-L1 expression by cancer cells has been implicated in cancer immune evasion, as binding of PD-1 on T cells to PD-L1 on tumor cells results in T cell exhaustion and apoptosis [2, 3]. Blocking of PD-1 binding to its ligand eliminates this negative feedback signal and prolongs anti-tumor immune activity [3]. PD-L1 expression in tumor tissues may have predictive value for PD-1 ICI therapy response in melanoma and other cancers [4, 5], although PD-L1 expression in the tumor tissue does not always correlate with PD-1 ICI therapy response. This may be due to dynamic changes in PD-L1 expression within the tumor tissue, and/or intra-patient heterogeneity in PD-L1 expression among tumor lesions [5]. These observations underscore the importance of understanding the mechanism how PD-L1 expression is regulated.

PD-L1 expression is regulated at multiple levels [6, 7]. At the genomic level, *PD-L1* locus amplification has been reported in B cell lymphoma, Hodgkin lymphoma and melanoma [6, 8]. At the transcriptional level, the pro-inflammatory cytokine IFNγ is a key inducer of *PD-L1* expression via the JAK/STAT [9] and NF-κB pathways [6], while the Toll-like receptor (TLR) induces *PD-L1* transcription through the MEK/ERK pathway [10, 11]. PD-L1 expression can be enhanced at the translational level by the eukaryotic translation initiation complex which stimulates STAT1 translation [7], or by oncogenic RAS which stabilizes *PD-L1* mRNA [7]. At the protein level, CMTM4 and CMTM6 prevent the ubiquitination and lysosomal degradation of the PD-L1 protein, thereby stimulating lysosomal recycling and increasing cell surface levels of PD-L1 [7].

Hypoxia is a hallmark of solid tumor development, due to an unstable tumor vasculature and a high metabolic rate [12]. Tumor hypoxia creates an immune suppressive tumor microenvironment, which hampers immunotherapy [13]. Hypoxia therefore represents a negative prognostic factor. Interestingly, recent studies suggest that hypoxia can induce PD-L1 expression in tumor cells [14–18] and may therefore promote tumor escape. The induction of PD-L1 expression by hypoxia has been reported in multiple primary and cancer cell types [14–18]. The hypoxia-induced transcription factors (HIF) are key regulators of the transcriptional response to hypoxia [12] and have been reported to transcriptionally upregulate PD-L1 expression in human renal cell carcinoma cell lines [14, 16], in the murine myeloid-derived suppressor cell line MSC-1 [17] and in the murine melanoma cell line B16 [15]. However, it is currently largely unknown whether hypoxia/HIF1 regulates PD-L1 expression in human melanoma. Moreover, it is not known whether hypoxia cooperates with IFNγ in regulating PD-L1 expression. The latter may be of particular importance as both are frequently present in the tumor microenvironment [9, 12]. We explored these questions using a panel of melanoma cell lines, and two publicly available RNAseq datasets of cutaneous melanoma.

## Methods

### TCGA data analysis

The R2 Genomics Analysis and Visualization platform (http://r2.amc.nl) was used to analyze the cutaneous melanoma dataset from the cancer genome atlas (TCGA) [8], and a recently published single-cell RNAseq melanoma dataset [19].

### Western blot

Cells were harvested and lysed as described before [20]. The protein concentration was determined using the Bradford assay (#39222, Serva, Heidelberg, Germany), and equal amounts were loaded on a gel. Protein levels were analyzed by Western blot as described before [21]. The following primary antibodies were used: mouse anti-human HIF1α (BD Biosciences, #610959), mouse anti-human PD-L1 (Cell Signaling Technology, #29122), mouse monoclonal β-Actin Antibody (C4) (#sc-47778). The following secondary antibodies were used: Donkey-anti-mouse IRDye 800CW (Li-cor Biosciences, Lincoln, NE.) and Donkey-anti-Rabbit IRDye 680CW (Li-cor Biosciences). All antibodies were diluted in 3% non-fat milk in TBST. Immunoblots were analyzed by Odyssey infrared imaging system (Li-cor Biosciences).

### Statistical analysis

All quantitative data are presented as the average ± standard deviation (SD), as compared to the indicated controls in at least three independent experiments. Statistical comparisons between two groups were performed using a two-tailed, independent t-test. Variances of two groups were compared with an F-test. Statistical comparisons between three or more groups were performed by analysis of variance (ANOVA, as indicated in the figures). Differences were considered significant with a p-value of < 0.05 (*p < 0.05, **p < 0.01, ***p < 0.001).

### RNA isolation, cDNA synthesis and quantitative PCR

RNA isolation and cDNA synthesis were performed as described before [20]. Quantitative PCR was performed according to MIQE standards [22]. Gene expression was calculated using the ΔΔCt method adapted for 2-reference gene correction (ACTB, RPS18) [20]. Oligonucleotides sequences used: human ACTB, RSP18 and BNIP3L [20], human *PD-L1* mRNA: 5′-TGAACTGACATGTCAGGCTG (forward), 5′-TACCACTCAGGACTTGATGG; human *HIF1α* mRNA: 5′-CATAAAGTCTGCAACATGGAAGGT-3′ (forward), 5′-ATTTGATGGGTGAGGAATGGGTT-3′.

### Cell culture and stimulation

Human melanoma cell lines MelAKR and MelJUSO and the murine melanoma cell line B16.F10 were cultured in RPMI 1640 (Gibco). The human melanoma cell lines MelWBO and Mel136.2 were cultured in IMDM (Gibco). The human melanoma cell line Mel88.23 and human cervical cancer cell line HeLa were cultured in DMEM (Gibco). All media were supplemented with 8% heat-inactivated fetal calf serum (FCS; #0270–106), 1% penicillin/streptomycin (P/S, #15140122) and 2 mM L-glutamine (#25030024, Thermofisher Scientific). All cell lines have been described before [21, 23, 24] and were cultured at 37 °C and 5% CO_2_, and at atmospheric oxygen levels (normoxia). For hypoxia, cells were incubated in the H35 Hypoxystation (Don Whitley Scientific) at 1% O_2_. One day prior to stimulation, cells were seeded in a total volume of 2 ml per well using 6-well plates (Greiner Bio One, #657160) at a cell density Mel88.23 (2.5 × 10^5^/well), Mel136.2, MelWBO and B16.F10 at 1 × 10^5^/well, MelAKR, MelJUSO, HeLa at 1.5 × 10^5^/well. Cells were exposed to hypoxia, incubated with IFNγ (Roche, #11040596001) or HIF-stabilizing agent DFO (Deferoxamine mesylate salt, Sigma-Aldrich, D9533), or a combination thereof, as indicated in the results. DFO-containing medium was replaced after 24 h.

### Luciferase reporter assay

Reporter assays were performed as described before [20] with the following modifications: melanoma cells were seeded at 1 × 10^4^/well (24-well plate, Greiner Bio One) one day prior to transfection (SuperFect Transfection Reagent, Qiagen). Per well 500 ng reporter plasmid, 20 ng TK renilla (used for normalization) and 200 ng expression plasmid were used for transfection. After four hours, the medium was replaced and cells were cultured in hypoxia or normoxia and incubated overnight, followed by measuring reporter activity using the Dual-Luciferase Reporter Assay (Promega) in a GloMax Discover System (Promega). The wildtype 952 bp human *PD-L1* promoter (pGL3 Basic) was obtained from Antoni Ribas [9]. The -1177/-933 *VEGFA* promoter was derived from the 1173 bp *VEGFA* promoter [25].

### Flow cytometry

For FACS analysis, adherent cells were washed twice with PBS and harvested using 1 × EDTA (2 mM). Cells were resuspended in FACS buffer (PBS + 0.05% Sodium Azide + 1% Bovine Serum Albumin) and transferred to a 96-well plate (Costar) on ice. Cells were washed twice in FACS buffer by centrifugation. Cells were incubated with PD-L1 antibody (PDL1-APC, Invitrogen (#17–5983-42), diluted 1:40–1:80) in FACS buffer for 20 min on ice in the dark. Both cell surface and intracellular PD-L1 stainings were performed to detect more immediate changes in PD-L1 protein expression, as differences in protein expression may be present intracellularly, but may not yet be detectable at the cell surface. Intracellular staining was performed by fixation of the cells in 200 µl fixation buffer (420801, biolegend) for 20 min at RT in the dark, followed by washing in permeabilization wash buffer (#421002, Biolegend, 1:10 diluted). PD-L1 staining was performed in permeabilization wash buffer for 30 min at RT in the dark. Cells were subsequently washed once in permeabilization wash buffer and resuspended in FACS buffer for acquisition. Unstained negative controls were included per stimulation for each cell line. Addition of 5 µl 7AAD (#00–6993-50, ebioscience) 5 min prior to measurement was used to determine cell viability. Cells were acquired by flow cytometry using on a BD FACS Canto II. Data were analyzed using FlowJo software (V6, Treestar). Per condition and cell line, the MFI of the cells in the live gate was analyzed, relative to the MFI of unstained cells to compensate for autofluorescence. FACS data represent the corrected-mean fold change MFI in hypoxia, relative to untreated control cells in normoxia.

### Lentiviral transduction with pLKO short hairpin RNA HIF1α vectors

HEK293T cells were seeded in 10 ml complete DMEM medium using 10 cm dishes coated with poly-L-lysine (0.01 mg/ml; Sigma-Aldrich). On the next day, cells were transfected according to the calcium phosphate transfection protocol. In brief, the viral vectors pMD2G (2.25 µg), pRRE (2.75 µg) and pRSV/REV (1.89 µg) were combined with 8 µg pLKO plasmid DNA and added to 500 μL 0.25 M CaCl_2_. Next, 500 μL 2 × HEPES-buffered saline (140 mM NaCl, 1.5 mM Na2HPO4, 50 mM HEPES, pH 7.05) was added (dropwise during vortexing at low speed). After 15 min, the mixture was added to the cells. On the next day, the medium was replaced. After 24 h, the virus-containing supernatant was filtered (0.22 µM filter, Millex) and added to the target cells. After 24 h, the medium was replaced with selection medium (medium with 2 µg/ml puromycine (Cayman chemical)). The following shRNA plasmids (MISSION shRNA vector database (Sigma-Aldrich)) were used: the control plasmid DNA pLKO1 SHC002 and three HIF-1α targeting constructs (TRNC 3808 (A6), 3809 (A7) or 3811 (A9).

## Results

### PD-L1 expression positively correlates with HIF1-signaling pathway expression in human melanoma

**Fig. 1:**
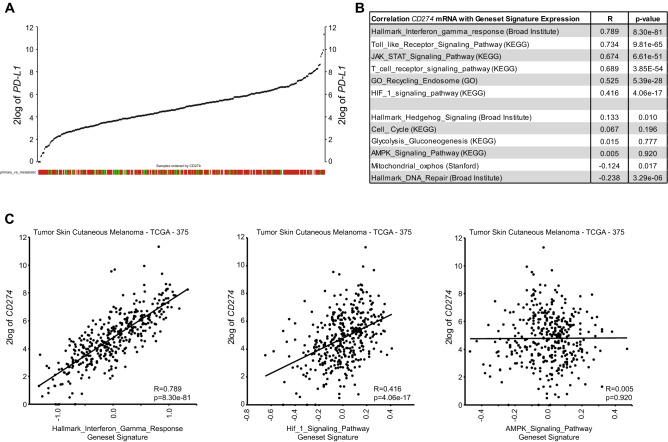
PD-L1 expression correlates with HIF1-signaling pathway expression in human melanoma. (**A**) YY-plot showing *PD-L1* (*CD274*) mRNA levels in 375 tumors from cutaneous melanoma patients (TCGA data). Red boxes indicate metastatic tumors; green boxes: primary tumors; and light gray boxes: not determined. (**B**) Table showing the correlation (R, correlation coefficient) and statistical significance (p-value) of *PD-L1* mRNA expression with expression of indicated geneset signatures in the TCGA melanoma data. (**C**) XY-plots showing three examples from (**B**)

To investigate the potential role of hypoxia in the regulation of PD-L1 expression in human melanoma, we made use of the cutaneous melanoma dataset from TCGA [8]. Analysis of *PD-L1* mRNA expression among 375 melanoma samples revealed a wide range of *PD-L1* expression levels in tumors (Fig. 1A), indicative of heterogeneity of PD-L1 expression in melanoma. *PD-L1* expression significantly correlated with the IFNγ/JAK/STAT pathway, a key regulator of PD-L1 expression [9] (Fig. 1B). As tumor-reactive immune cells are thought to be a main source of IFNγ in the tumor microenvironment, *PD-L1* mRNA expression also significantly correlated with the lymphocyte score (Supplementary Figure S1A). However, *PD-L1* mRNA expression did not significantly correlate with the type of metastasis, or any of the previously identified genomic melanoma subtypes (Supplementary Figure S1B) [8]. Other pathways known to be involved in the regulation of *PD-L1* expression, like the Toll-like receptor (TLR) pathway [10, 11], and lysosomal recycling through CMTM proteins [7], also significantly correlated with *PD-L1* expression (Fig. 1B). *PD-L1* mRNA expression also significantly correlated with the HIF1-signaling geneset signature, although to a lesser extent (Fig. 1B, C). Other geneset signatures, *e.g.* AMPK signaling, did not correlate with PD-L1 expression (Fig. 1B, C). Gene-to-gene correlations also revealed a strong correlation between *PD-L1* and *IFN*γ or *STAT1* expression, whereas a weaker correlation was observed between *PD-L1* and *HIF1α* (Supplementary Figure S1C-E). Nonetheless, the correlation between *HIF1α* and *PD-L1* was comparable to *HIF1α* and its canonical target genes *BNIP3L* and *PDK1* (Supplementary Figure S1F). These data suggest a role for hypoxia in the regulation of PD-L1 expression in melanoma.

### Hypoxia differentially affects PD-L1 expression in a panel of human melanoma cell lines

**Fig. 2:**
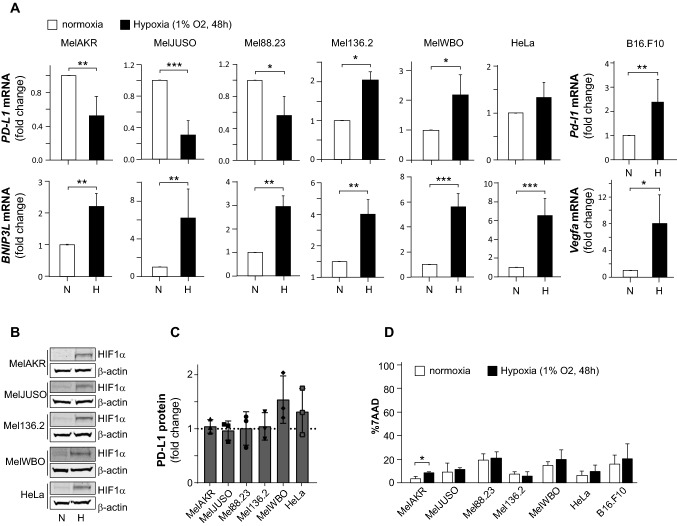
Hypoxia differentially affects PD-L1 expression in melanoma cells. (**A**) The human melanoma cell lines MelAKR, MelJUSO, Mel88.23, Mel136.2, MelWBO, the mouse melanoma cell line B16.F10 and the human cervical cancer cell line HeLa were cultured for 48 h in normoxia or hypoxia, after which cells were harvested and *PD-L1* mRNA expression was analyzed by qPCR. Graphs show *PD-L1* and *BNIP3L* mRNA levels depicted as fold change, as compared to normoxia. White bars present normoxic (N) and black bars hypoxic (H) conditions. The HIF1 target *BNIP3L* (and *Vegfa* in the mouse B16.F10 cells) were used as markers of HIF1 activity, and (**B**) Western Blots showing HIF1α protein levels for the indicated cell lines. β-ACTIN protein levels serve as loading control. (**C**) Total PD-L1 protein levels (both intracellular and extracellular) were determined in human cell lines by FACS under the same conditions as in (A). Graphs indicate the fold change in PD-L1 protein levels in hypoxia, as compared to normoxia. (**D**) Cell viability was analyzed for all cell lines in normoxia and hypoxia. All quantified data are presented as the average ± standard deviation, as compared to the control (N) in at least three independent experiments. Statistical significance of differences between normoxia and hypoxia was tested. *p < 0.05, **p < 0.01, ***p < 0.001

Next, we analyzed the potential hypoxic regulation of PD-L1 expression in a panel of in human melanoma cell lines and the mouse melanoma B16 cell line. The cervical cancer HeLa cell line was also analyzed in parallel, serving as a hypoxia-responsive reference cell line, as we used it previously to investigate hypoxia-regulated gene expression [20]. At the mRNA level, the expression of the canonical HIF1 target *BNIP3L* was induced under hypoxic conditions in all human cell lines, indicating HIF1 activation (Fig. 2A). Likewise, in the murine B16 cells the canonical HIF1 target *Vegfa* was analyzed and induced. Hypoxic HIF1 activation was also confirmed by HIF1α protein accumulation (Fig. 2B). Hypoxia induced *PD-L1* expression in murine melanoma B16 cells, as previously described [15, 17]. However, we observed that hypoxia either repressed or induced *PD-L1* mRNA expression in human melanoma cells (Fig. 2A). This effect was, however, not confirmed at the protein level (Fig. 2C). The hypoxic changes in *PD-L1* mRNA expression were not related to indirect effects of hypoxia on cell viability as this was not affected (Fig. 2D). In conclusion, our study reveals that hypoxia can either stimulate or repress *PD-L1* mRNA expression in human melanoma cells.

### HIF1 has a minor effect on PD-L1 expression in response to hypoxia or the hypoxia mimetic DFO

**Fig. 3:**
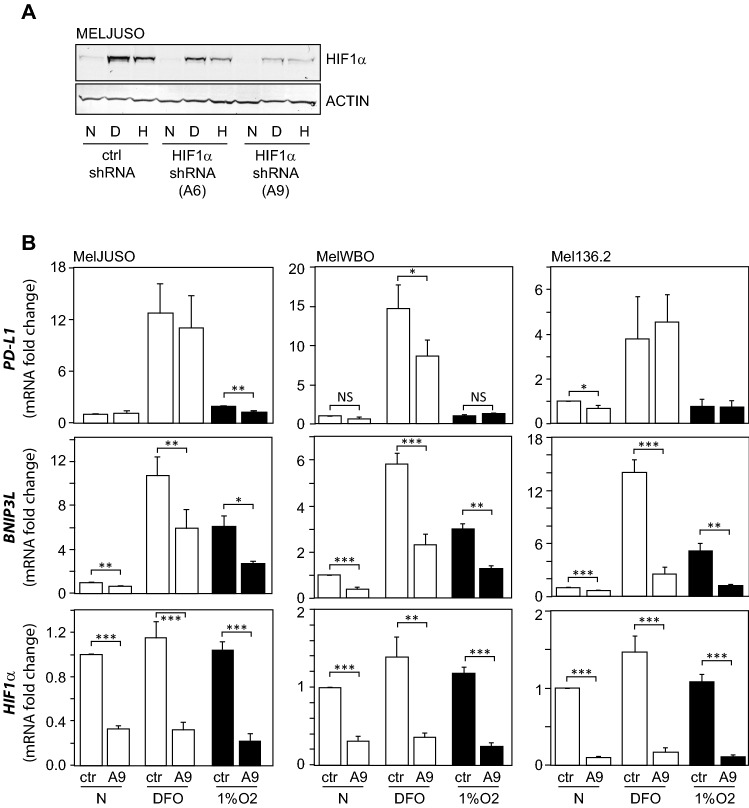
A minor role for HIF1 in PD-L1 regulation in response to hypoxia or DFO. (**A**) MELJUSO cells were transduced with lentiviral shRNA vector A6 or A9 targeting HIF1α, or a control vector (ctrl). Transduced cells were cultured for 24 h in normoxia (N, white bars), hypoxia (H, 1% O_2_, black bars) or in the presence of 100 μM DFO (D). HIF1α protein levels were determined by Western blot. Actin protein levels serve as loading control. (**B**) MelJUSO, MelWBO and Mel136.2 cell lines transduced with the lentiviral shRNA vector A9 targeting HIF1α, or a control vector, were stimulated with DFO (100 μM) or hypoxia (1% O_2_) for 24 h, after which cells were harvested. *HIF1α*, *BNIP3L* and *PD-L1* mRNA levels were determined by qPCR. White bars present normoxic (N), and black bars hypoxic conditions

To verify a role for HIF1 in the hypoxic mRNA regulation of PD-L1 expression in melanoma cells, we tested whether HIF1α knockdown would affect *PD-L1* expression. To achieve this goal, we first validated two shRNA vectors targeting HIF1α (A6 or A9) in MelJUSO cells. Hypoxia and DFO induced HIF1α protein expression in MelJUSO cells after 24 h, which was most efficiently reduced by the HIF1α targeting shRNA A9 (Fig. 3A). Next, we transduced several melanoma cell lines with the HIF1α targeting vector A9 or a control hsRNA and stimulated the cells for 24 h with 100 μM DFO or 1% O_2_ hypoxia. The MelJUSO, MelWBO and Mel136.2 cell lines were selected for further experiments as the HIF1α targeting vector A9 significantly reduced HIF1α expression in these cell lines (Fig. 3B, lower panel). This resulted in a functional knockdown, demonstrated by significantly reduced expression of the HIF1 target *BNIP3L* (Fig. 3B, middle panel). Although DFO significantly induced *PD-L1* expression, this occurred independent of HIF1α in MelJUSO and Mel136.2 cells, and partly HIF1α-dependent in MelWBO cells (Fig. 3B). In hypoxia, HIF1α-knockdown reduced hypoxic PD-L1 expression in MelJUSO cells, but not in MelWBO and Mel136.2 cells (Fig. 3B). These data indicate a minor role for HIF1 in the regulation of *PD-L1* gene expression.

### IFNγ-mediated stimulation of PD-L1 expression is affected by hypoxia

**Fig. 4:**
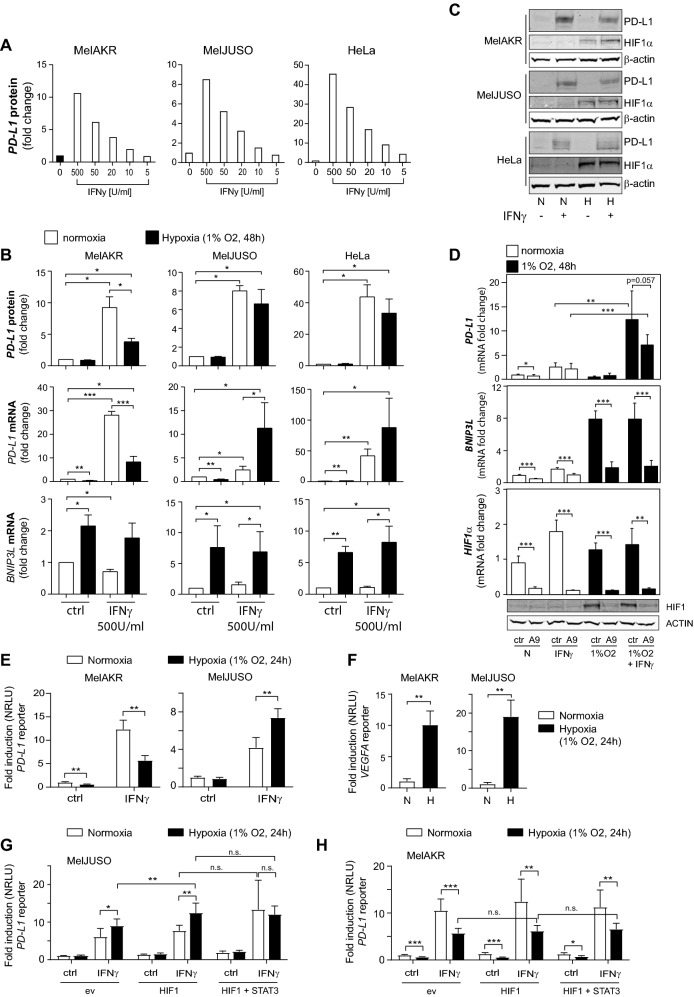
Stimulation of PD-L1 expression by IFNγ is affected by hypoxia. (**A**) The MelAKR, MelJUSO and HeLa cells were incubated in normoxia for 48 h with different concentrations of IFNγ, ranging from 500 to 5 U/ml, as indicated. Cells were harvested, and PD-L1 protein expression at the cell surface was analyzed using flow cytometry. Data represent median fluorescent intensity (MFI) of PD-L1 staining, as compared to untreated cells (ctrl), per experiment. (**B**) MelAKR, MelJUSO and HeLa cells were seeded one day prior to stimulation for 48 h with IFNγ (500 U/ml) in normoxia (N, white bars) or hypoxia (H, 1% O_2_, black bars). *PD-L1* and *BNIP3L* mRNA levels were determined by qPCR, and PD-L1 protein levels (surface expression) by flow cytometry. Graphs show the fold change in MFI of PDL1-staining, as compared to normoxia. (**C**) Western blot analysis of HIF1α and PD-L1 protein levels under same conditions as described in (B). β-ACTIN protein levels serve as loading control. (**D**) MELJUSO cells transduced with the HIF1α targeting shRNA vector A9 or a control (ctrl) were cultured for 48 h in normoxia (N) and incubated with IFNγ (500U/ml), hypoxia (1% O_2_) or a combination thereof, as indicated. The fold change in mRNA expression was determined compared to ctrl cells in N. All quantitative data are presented as the average ± standard deviation of 5 independent experiments. Statistical significance of differences between ctrl and A9 was analyzed for all conditions, and for *PD-L1* also between the IFNγ and IFNγ plus hypoxia conditions. The lower panels indicate Western blot analysis of HIF1α and β-ACTIN (loading control) protein levels for the indicated conditions. (**E**) *PD-L1* reporter assay in MelAKR and MelJUSO cells. Cells were incubated with IFNγ (500U/ml), hypoxia or a combination thereof, for 24 h, as indicated. Graphs show the fold induction of normalized relative luciferase units (NRLU), as compared to normoxia. (**F**) *VEGFA* reporter assay under the same conditions as in (E). *PD-L1* reporter assay in MelJUSO (**G**) and MelAKR (**H**) as described in (E) but now in combination with HIF1, or HIF1 and STAT3 overexpression. All quantitative data are presented as the average ± standard deviation in at least three independent experiments. *p < 0.05, **p < 0.01, ***p < 0.001

IFNγ and hypoxia are both present in the tumor microenvironment [9, 12] and can each induce *PD-L1* expression. Therefore, we next explored whether hypoxia could affect the IFNγ-induced PD-L1 expression. For these analyses, we used MelAKR and MelJUSO cell lines (in addition to HeLa cells), as hypoxia alone did not affect PD-L1 expression in these cell lines (Fig. 2C). IFNγ-induced PD-L1 protein expression at the cell surface in a dose-dependent manner (Fig. 4A, Supplementary Figure S2A). Next, the cells were stimulated with hypoxia, IFNγ or a combination thereof. Hypoxia induced HIF1α protein, and *BNIP3L* target gene expression under these condition, indicating HIF1 activation (Fig. 4B, C). In the presence of both hypoxia and IFNγ, hypoxia attenuated IFNγ-mediated induction of *PD-L1* mRNA expression in MelAKR cells, whereas hypoxia enhanced the induction of *PD-L1* mRNA expression by IFNγ in MelJUSO and HeLa cells (Fig. 4B). The inhibitory effect of hypoxia on the induction of PD-L1 expression by IFNγ in MelAKR cells was confirmed at the protein level by FACS (Fig. 4B) and by western blot (Fig. 4C). However, the co-stimulatory effect of hypoxia and IFNγ on *PD-L1 mRNA* expression did not result in enhanced PD-L1 protein expression in MelJUSO and HeLa cells. Therefore, we next tested whether a more potent HIF1 stimulus would enhance PD-L1 protein expression in IFNγ-stimulated MelJUSO cells. For this purpose, we used the hypoxia mimetic DFO, which more strongly induces HIF1α protein, and *BNIP3L* mRNA expression than hypoxia (Fig. 3A, B). In contrast to hypoxia (Fig. 4B), DFO by itself significantly enhanced PD-L1 protein expression (Supplementary Figure S2B and S2C). DFO also enhanced IFNγ-mediated PD-L1 protein expression in MelJUSO cells as quantified by FACS (Supplementary Figure S2B). Next, we verified a potential role for HIF1 in the co-stimulatory effect of hypoxia and IFNγ on PD-L1 expression in MelJUSO cells (Fig. 4B). For this, MelJUSO cells were transduced with the HIF1α A9 or control shRNA (Fig. 3) and exposed to IFNγ and hypoxia. Although the HIF1α targeting vector significantly reduced *HIF1α* and *BNIP3L* expression under all conditions (Fig. 4D), HIF1α knockdown did not reduce (*p* = 0.057) the co-stimulatory effect of hypoxia and IFNγ on *PD-L1* expression (Fig. 4D).

Next, we performed promoter studies to investigate whether the differential effect of hypoxia on IFNγ-induced PD-L1 expression (Fig. 4B) is controlled at the *PD-L1* promoter. Similar to the effect on mRNA expression (Fig. 4B), hypoxia repressed IFNγ-induced *PD-L1* promoter activation in MelAKR cells, while hypoxia stimulated this in MelJUSO cells (Fig. 4E). This demonstrates that hypoxia differentially controls IFNγ-induced *PD-L1* expression at the promoter level. Because HIF1 was activated in both cell lines in hypoxia, as the HIF1-target promoter *VEGFA* was significantly induced (Fig. 4F), the differential effect of hypoxia on IFNγ-stimulation of *PD-L1* expression cannot be explained by whether or not HIF1 is activated. Therefore, we also explored whether the differential effect of hypoxia on *PD-L1* mRNA expression (Fig. 4B) was due to co-regulation by other transcription factors, *e.g*. STAT3. It has been reported that STAT3 can interact with HIF1 [18, 26], and that a complex of STAT3 and HIF1 induced PD-L1 expression in colon cancer cells [27], and in hypoxic NSCLC cells [18]. However, although HIF1 overexpression significantly enhanced IFNγ-induced *PD-L1* expression in hypoxia in MelJUSO cells, STAT3 did not further enhance this (Fig. 4G). Moreover, STAT3 overexpression did not prevent the hypoxic repression of IFNγ-induced PD-L1 expression in MelAKR cells (Fig. 4H). Analysis of the 952 bp *PD-L1* promoter fragment did not reveal the presence of consensus HIF1 binding sites (RCGTG [25]), and therefore, HIF1 probably indirectly controls the *PD-L1* promoter. This is not unlikely as STAT factors also indirectly control the 952 bp *PD-L1* promoter fragment [9].

### PD-L1 expression correlates with HIF1α expression within melanoma sub-populations

**Fig. 5:**
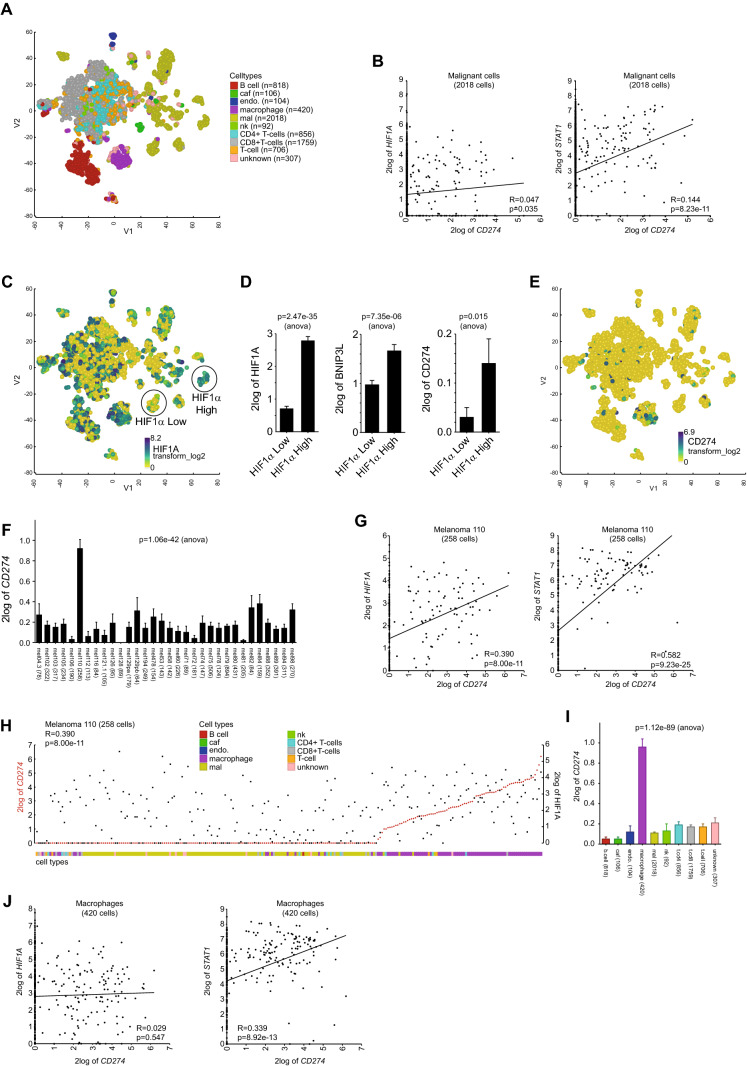
Single-cell analysis reveals a positive correlation between HIF1α and PD-L1 expression in melanoma subpopulations with high HIF1α levels, or in tumors with elevated PD-L1 levels. (**A**) T-stochastic neighbor embedding plot (t-SNE, perplexity = 50) using single-cell RNAseq analysis of 7186 cells from 33 melanoma tumors [19]. CAF (cancer-associated fibroblasts), endo (endothelial cells), mal (malignant cells), nk (natural killer cells). (**B**) XY-plots showing the correlation (R) between *PD-L1* (*CD274*) and *HIF1α* or *STAT1* mRNA expression (log2 transformed) in the subset of 2018 melanoma cells. (**C**) *HIF1α* expression in the t-SNE plot from (A). Melanoma subpopulations with low (HIF1α Low), or high (HIF1α High) *HIF1α* expression. (**D**) Graphs show *HIF1α*, *BNIP3L* and *PD-L1* expression in the HIF1α low and high melanoma cell subpopulations. (**E**) *PD-L1* expression in the t-SNE plot from (A). (**F**) Graph depicting *PD-L1* expression in all 33 melanoma samples. (**G**) XY-plots showing the correlation (R) between *PD-L1* (*CD274*) and *HIF1α* or *STAT1* mRNA expression in tumor 110 (258 cells) (**H**) YY-plot showing HIF1α (black squares) and PD-L1 (red dots) in tumor 110. Cells were ordered by *CD274*. (**I**) Bar graph showing *PD-L1* (*CD274*) expression in the different cell types. (**J**) XY-plots showing the correlation (R) between *PD-L1* (*CD274*) and *HIF1α* or *STAT1* mRNA expression in macrophages (420 cells) from all tumors

We further explored the correlation between *PD-L1* and *HIF1α* expression in subpopulations of melanoma cells, using the recently published single-cell RNA-sequencing (RNAseq) data of 33 melanoma tissues [19]. RNAseq analysis of 7186 cells by t-stochastic neighbor embedding (t-SNE) revealed multiple melanoma-cell subpopulations, and various types of stromal and immune cell populations (Fig. 5A), as shown previously [19]. PD-L1 expression did not significantly correlate with *HIF1α* in the total population of melanoma cells (Fig. 5B), although it correlated with expression of the HIF1-pathway gene-signature (Supplementary Figure S3A). However, both expression of *STAT1* (Fig. 5B) and the JAK/STAT-pathway gene-signature (Supplementary Figure S3A) displayed a stronger correlation with *PD-L1* expression. Next, we analyzed whether *PD-L1* expression correlated with *HIF1α* expression in subpopulations of malignant cells. Among melanoma subpopulations, varying levels of *HIF1α* expression were observed (Fig. 5C). A subpopulation with high *HIF1α* expression not only showed significantly higher expression of the canonical HIF1 target *BNIP3L*, but also of *PD-L1* (*CD274*), as compared to a melanoma subpopulation with low *HIF1α* expression (Fig. 5D). This suggests that HIF1 may stimulate PD-L1 expression in malignant subpopulations where HIF1 expression levels are elevated.

### High expression of PD-L1 in tumor-associated macrophages (TAMs) in melanoma

*PD-L1* expression was generally low in the melanoma cells (Fig. 5E). Therefore, we next analyzed *PD-L1* expression among the 33 melanoma tissues. This revealed a relatively high *PD-L1* expression in tumor 110 (Fig. 5F). In the 258 cells analyzed from this tumor tissue, *PD-L1* expression correlated significantly with *HIF1α* expression, although *PD-L1* expression correlated stronger with *STAT1* (Fig. 5G). Similarly, *PD-L1* expression also correlated stronger with the JAK/STAT-pathway gene-signature, as compared to the HIF1-pathway gene signature (Supplementary Figure S3A). Surprisingly, we found that *PD-L1* expression in tumor 110 appeared to be primarily elevated in TAMs (Fig. 5H). Notably, PD-L1 expression was significantly higher in TAMs from all 33 tumors (Fig. 5I). However, comparable to the melanoma cells, *HIF1α* (or the HIF1-pathway gene-signature) did not significantly correlate with *PD-L1* expression in TAMs, while STAT1 (or the JAK/STAT-pathway gene-signature) did (Fig. 5J, Supplementary Figure S3A). These data indicate that HIF1 may contribute to PD-L1 expression in TAMs from tumors with elevated PD-L1 levels.

## Discussion

Little evidence is available until present on the transcriptional control of PD-L1 expression by HIF factors in human cancer. For example, although two studies reported a positive correlation between PD-L1 expression and HIF target gene expression in clear cell renal cell carcinoma patients [14, 16], this was not confirmed in a third study [28]. Our analysis of two RNAseq datasets of human cutaneous melanoma revealed a positive correlation between *HIF1α* (or a geneset signature of the HIF1 signaling pathway) and *PD-L1* expression (Figs. 1B, 5D, G). Using a recently published single-cell RNAseq dataset, we furthermore show that this positive correlation occurs either in melanoma subpopulations with a high *HIF1α* expression (Fig. 5D), or in tumors that with a high *PD-L1* expression (Fig. 5G). This suggests that HIF1 may contribute to *PD-L1* expression in specific melanoma subpopulations with high HIF1 expression (Fig. 5C, D), or in PD-L1 positive tumors (Fig. 5F, G). In addition, analysis of PD-L1 expression in a panel of melanoma cell lines confirmed the hypoxic induction of *Pd-l1* mRNA expression in murine B16 cells [15, 17], but also revealed that hypoxia differentially regulates (IFNγ-induced) *PD-L1* expression in a panel of human melanoma cell lines (Figs. 2A, 4B). The latter observation is in contrast to the primarily stimulatory role of hypoxia in the regulation of PD-L1 expression reported so far [14–18]. However, both the RNAseq data (Fig. 1B; Supplementary Figure S1C-E; Fig. 5D, G, J) and the in vitro data (Figs. 3B, 4B–E) indicate a secondary role for hypoxia/HIF1 in the regulation of PD-L1 expression in melanoma compared to the IFNγ/JAK/STAT pathway, the key regulator of PD-L1 expression.

The main inducer of PD-L1 expression in melanoma is therefore IFNγ. Since PD-L1 expression significantly correlated with tumor immune infiltration (Supplementary Figure S1A), IFNγ released from infiltrating immune cells is probably the main inducer of PD-L1 in the tumor microenvironment. This also underlines *PD-L1* mRNA expression in tumor tissue as a marker of immunogenicity [29]. In addition, we observed that PD-L1 is mainly expressed in TAMs in melanoma tissue (Fig. 5H, I). This is consistent with recent studies that describe an important role for PD-L1/PD1 signaling in TAMs. It was reported that PD-L1 signaling delivers a constitutively negative signal to macrophages, while inhibition of PD-L1 stimulates macrophage proliferation and activation [30]. Moreover, PD-L1 inhibition also enhances the tumor infiltration of macrophages and phagocytosis activity, resulting in tumor growth inhibition (among which melanoma) in an macrophage-dependent manner [30, 31]. The role of PD-L1 signaling at the level of TAMs may be more important than anticipated. However, our data also suggest that the JAK/STAT pathway, but not HIF1, likely drives PD-L1 expression in TAMs (Fig. 5J).

The more secondary role for hypoxia and HIF1 in the regulation of PD-L1 might be explained by the observation that hypoxia can either stimulate or repress *PD-L1* expression (Fig. 2A). A differential effect of HIF1 activation on PD-L1 expression was also observed in a panel of non-small cell lung cancer (NSCLC) cell lines [18]. We also observed in MelJUSO cells that although hypoxia by itself represses PD-L1 expression (Fig. 2A), it enhances IFNγ-induced PD-L1 expression (Fig. 4B). Likewise, HIF1 activation itself had little effect on PD-L1 expression in H23 NSCLC cells, while it significantly enhanced EML4-ALK-induced PD-L1 expression in these cells [18]. These data indicate that HIF1 alone may be insufficient to induce PD-L1 expression and possibly cooperates with other factors. We hypothesized that STAT3 could be such a cooperating factor. This was based on its role in promoting PD-L1 expression downstream of IFNγ in melanoma [9], the correlation between *STAT3* and *PD-L1* mRNA levels (Supplementary Figure S3B), the fact that STAT3 can interact with HIF1 [18, 26], and has been reported to cooperate with HIF1 on the stimulation of PD-L1 expression in colon cancer cells [27], and in hypoxic NSCLC cells [18]. However, our reporter data exclude STAT3 as a cooperating factor in the differential regulation of PD-L1 by hypoxia (Fig. 4G, H). Another factor that could hamper the hypoxic induction of PD-L1 may be a counteracting hypoxia-induced repressor. A repressive element has indeed been identified in the 952 bp *PD-L1* promoter fragment [9]. Although we recently identified E2F7 as a hypoxia-induced transcriptional repressor [20], no E2F7 consensus binding sites (TTCCCGCC, [25]) were identified in the 952 bp *PD-L1* promoter fragment, the region through which hypoxia differentially affects PD-L1 expression (Fig. 4E). This excludes E2F7 as an hypoxia-induced repressor that (directly) interferes with PD-L1 expression.

Our study overall suggests a secondary role for hypoxia and HIF1 in the regulation of *PD-L1* expression in melanoma. However, our study also indicates that hypoxia can affect IFNγ-induced *PD-L1* expression. Hypoxia could therefore play a role in tumor immune surveillance through the regulation of *PD-L1* expression in melanoma.

### Supplementary Information

Below is the link to the electronic supplementary material.

**Supplementary Figure S1**. Analyses of PD-L1 and HIF1-related gene expression in the cutaneous melanoma (TCGA data). (**S1A**) Boxplots showing the positive correlation between *PD-L1* expression and the *lymphocyte score*. The lymphocyte score is defined as the sum of the lymphocyte distribution and density scores (0-6), as described previously [8]. 0 indicates no tumor infiltration by lymphocytes, 6 the highest level of TILs. The number between parenthesis indicates the number of tumors per lymphocyte score. (**S1B**) Boxplots showing the variation of PD-L1 expression within different groups. The left plot shows the variation of *PD-L1* expression between primary and metastasized tumors. The middle plot shows the variation between tumor types classified as distant, lymph node and regional skin metastasis, and primary disease. The right plot display the variation of PD-L1 expression within the indicated genomic subtypes. The numbers between parenthesis indicates the number of tumors for that specific track. Abbreviations: R, correlation coefficient; p, p-value. (**S1C**) Graph showing the distribution of the correlation coefficient (R) of all genes (STAT1, IFNγ, HIF1α highlighted) in relation to *PD-L1* expression. For example, the expression of 134 genes (0.7% of all genes) have a correlation (R) strength of 0.7 with *PD-L1*. IFNγ is one of the 134 genes. (**S1D**) XY-plots showing the significant correlation of two genes (*STAT1* and *IFNγ*) with *PD-L1*. (**S1E**) Correlation (R) between *HIF1α* and *PD-L1* mRNA levels in 375 cutaneous melanoma samples. (**S1F**) correlation of *HIF1α* with several of its canonical target genes (*BNIP3L*, *PDK1*). **Supplementary Figure S2**. Regulation of PD-L1 expression by starvation or the hypoxia mimetic DFO. (**S2A**) MelAKR, MelJUSO and HeLa cells were incubated with 500 or 50U/ml IFNγ, or were left untreated in normoxia for 48 hour, as indicated. Cells were harvested and PD-L1 protein expression at the cell surface was analyzed by flow cytometry. Data represent MFI of PD-L1-staining, as compared to untreated cells (ctrl). Quantitative data are presented as the average ± standard deviation in at least three independent experiments. *p<0.05, **p<0.01, ***p<0.001. (**S2B**) DFO induces *PD-L1* mRNA and protein expression in melanoma cells. Cells were seeded one day prior to incubated with DFO (50µM), IFNγ (500U/ml) or a combination of DFO and IFNγ, for 48 hours, as indicated. DFO was refreshed after 24h. Cells were harvested and processed for mRNA (*BNIP3L*, *PD-L1*) and protein (PD-L1) analysis using qPCR and flow cytometry, respectively. (**S2C**) Western blot analysis of HIF1α and PD-L1 protein levels in melanoma cells incubated with DFO (50µM) and/or IFNγ (500U/ml) for 48h, as indicated. Similar amounts of protein were loaded as determined by Bradford and β-ACTIN staining. Quantitative PCR data represent the mean of at least three independent experiments ± standard deviation. PD-L1 protein expression data are presented as MFI of PDL1-staining, for at least three independent experiments. Fold change was calculated compared to unstimulated cells. *p<0.05, **p<0.01, ***p<0.001. **Supplementary Figure S3**. Analysis of gene-signature and STAT3 expression in relation to PD-L1 expression in melanoma. (**S3A**) Table showing the correlation (R, correlation coefficient) and statistical significance (p-value) of *PD-L1* mRNA expression with expression of indicated geneset signatures in malignant cells, tumor 110, or in macrophages, using the single-cell RNAseq dataset from 33 melanoma tumors [19]. (**S3B**) XY-plot showing a significant correlation between *STAT3* and *PD*-L1 mRNA expression in 375 cutaneous melanoma samples from TCGA. (PDF 358 kb)

## Data Availability

The skin cutaneous melanoma dataset is publicly available through The Cancer Genome Atlas data portal (https://portal.gdc.cancer.gov/). The R2 platform for Genomics Analysis and Visualization is available from http://r2.amc.nl.

## Abbreviations

bp: Basepair
MFI: Median fluorescent intensity
PD-L1: Programmed cell death ligand 1
PD-1: Programmed cell death 1
SD: Standard deviation
TAMs: Tumor-associated macrophages
TCGA: The cancer genome atlas
TLR: Toll-like receptor
