# Supplementary material for: A secondary role for hypoxia and HIF1 in the regulation of (IFNγ-induced) PD-L1 expression in melanoma
Source: Cancer Immunol Immunother. 2021 Jul 15;71(3):529–40. doi: 10.1007/s00262-021-03007-1 (PMC8854324; doi:10.1007/s00262-021-03007-1)

Supplementary Figure S1

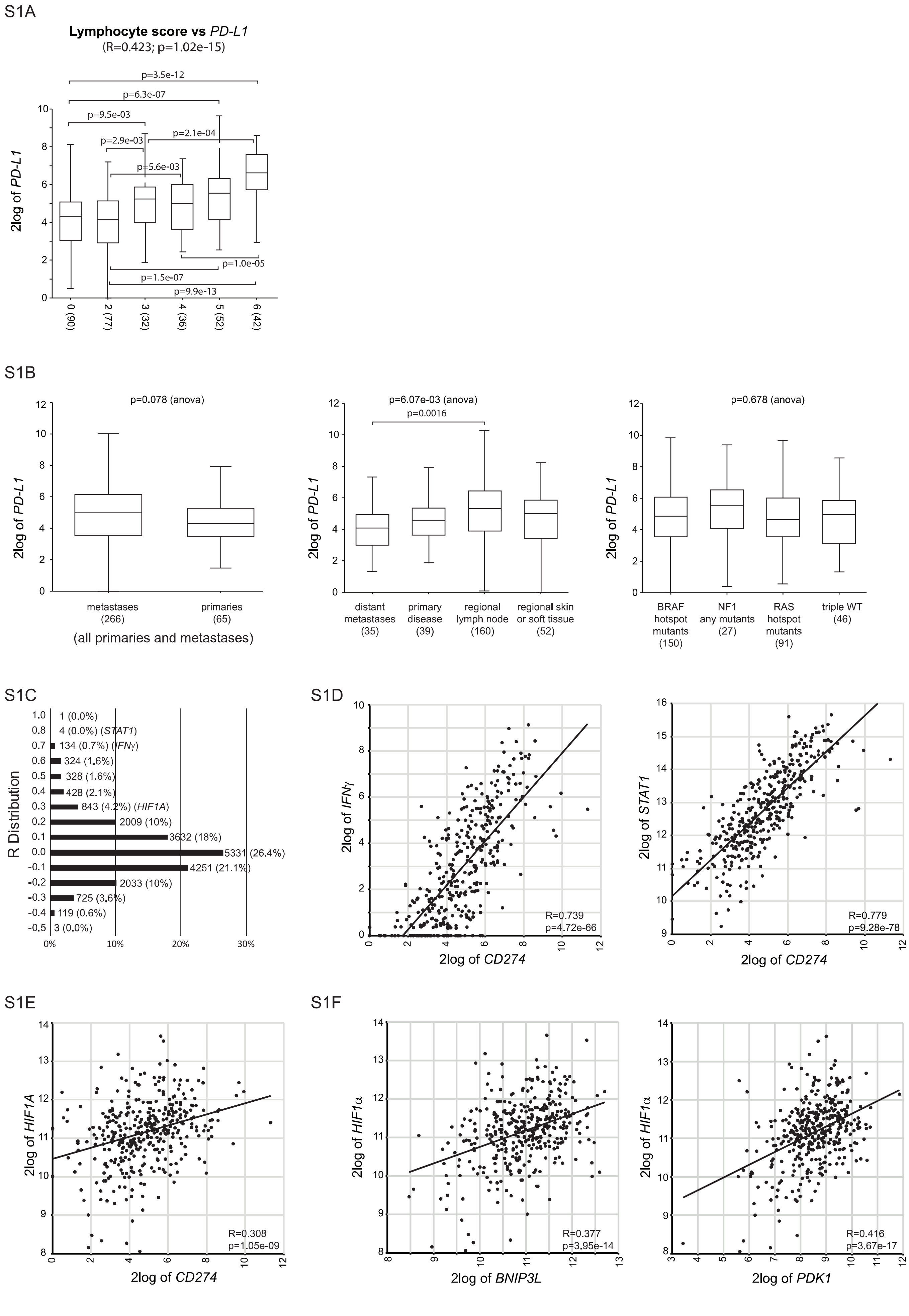

Supplemental Figure S2

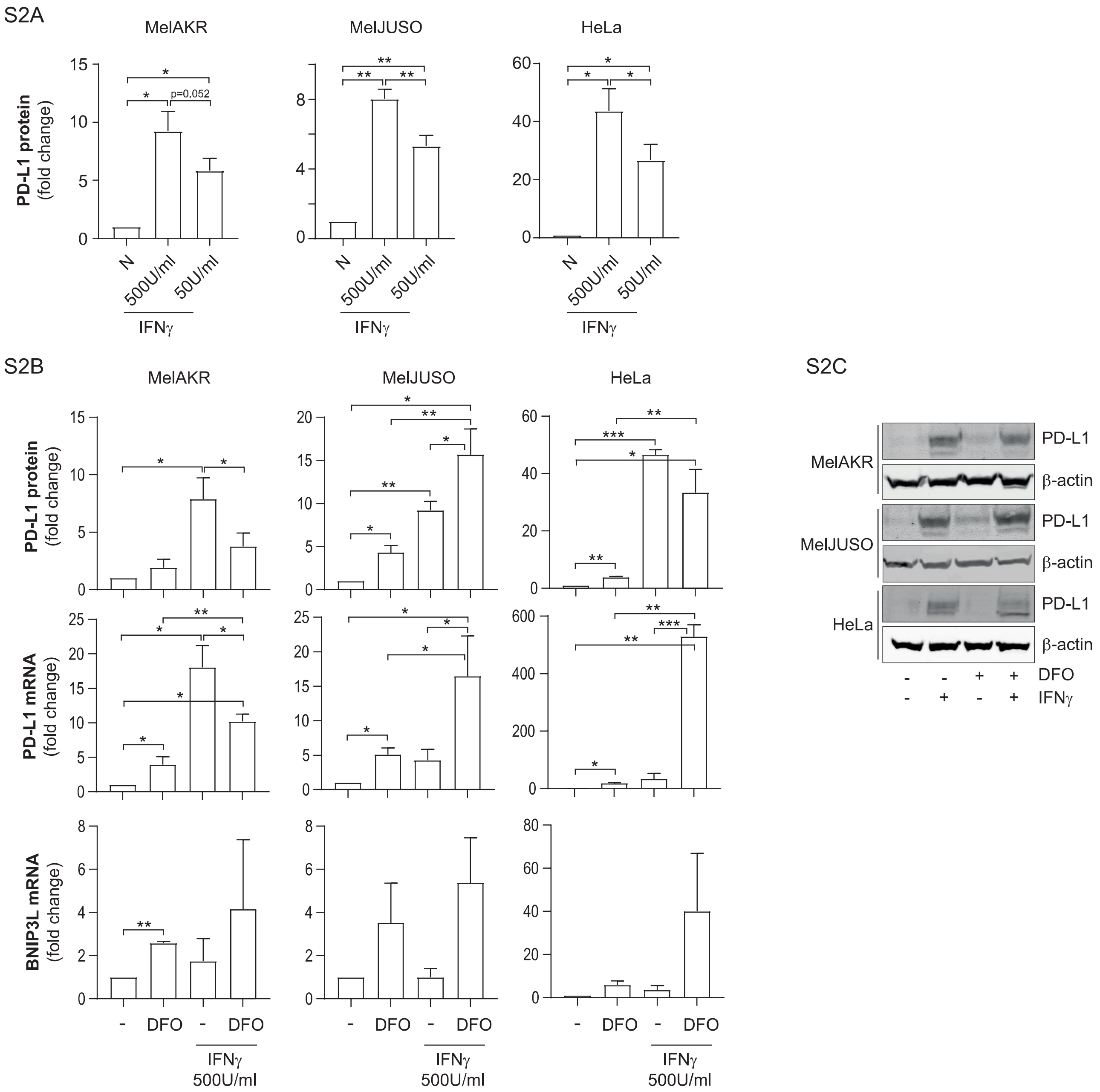

Supplemental Figure S3

S3A

**Correlation *CD274* mRNA with Geneset Signature Expression**	**R**	**p-value**
**Melanoma cells** (2018 cells)		
JAK_STAT_Signaling_Pathway (KEGG)	0.169	2.47e-14
HIF_1_signaling_pathway (KEGG)	0.119	7.79e-08
**Tumor 110** (258 cells)		
JAK_STAT_Signaling_Pathway (KEGG)	0.537	1.03e-20
HIF_1_signaling_pathway (KEGG)	0.271	9.85e-06
**Macrophage** (420 cells)		
JAK_STAT_Signaling_Pathway (KEGG)	0.122	0.012
HIF_1_signaling_pathway (KEGG)	0.031	0.521

S3B

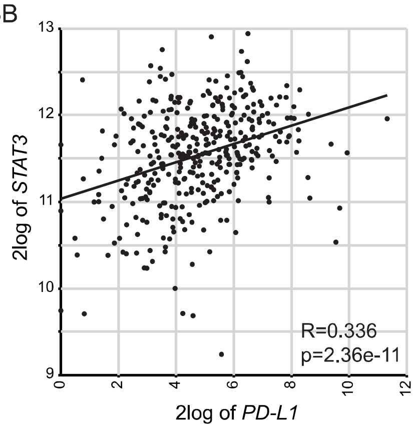

Supplement: Supplementary file 1 — Supplementary Figure S1. Analyses of PD-L1 and HIF1-related gene expression in the cutaneous melanoma (TCGA data). (S1A) Boxplots showing the positive correlation between PD-L1 expression and the lymphocyte score. The lymphocyte score is defined as the sum of the lymphocyte distribution and density scores (0-6), as described previously [8]. 0 indicates no tumor infiltration by lymphocytes, 6 the highest level of TILs. The number between parenthesis indicates the number of tumors per lymphocyte score. (S1B) Boxplots showing the variation of PD-L1 expression within different groups. The left plot shows the variation of PD-L1 expression between primary and metastasized tumors. The middle plot shows the variation between tumor types classified as distant, lymph node and regional skin metastasis, and primary disease. The right plot display the variation of PD-L1 expression within the indicated genomic subtypes. The numbers between parenthesis indicates the number of tumors for that specific track. Abbreviations: R, correlation coefficient; p, p-value. (S1C) Graph showing the distribution of the correlation coefficient (R) of all genes (STAT1, IFNγ, HIF1α highlighted) in relation to PD-L1 expression. For example, the expression of 134 genes (0.7% of all genes) have a correlation (R) strength of 0.7 with PD-L1. IFNγ is one of the 134 genes. (S1D) XY-plots showing the significant correlation of two genes (STAT1 and IFNγ) with PD-L1. (S1E) Correlation (R) between HIF1α and PD-L1 mRNA levels in 375 cutaneous melanoma samples. (S1F) correlation of HIF1α with several of its canonical target genes (BNIP3L, PDK1). Supplementary Figure S2. Regulation of PD-L1 expression by starvation or the hypoxia mimetic DFO. (S2A) MelAKR, MelJUSO and HeLa cells were incubated with 500 or 50U/ml IFNγ, or were left untreated in normoxia for 48 hour, as indicated. Cells were harvested and PD-L1 protein expression at the cell surface was analyzed by flow cytometry. Data represent MFI of PD-L1-stain [file 262_2021_3007_MOESM1_ESM.pdf]
